# Double Vision: Isolated Third Cranial Nerve Palsy After Cardiac Catheterization

**DOI:** 10.7759/cureus.9202

**Published:** 2020-07-15

**Authors:** Mohammad H Jilani, Hameed Iqbal, Syed Huda, Ali Younas Khan, Larry Charlamb

**Affiliations:** 1 Internal Medicine, State University of New York Upstate Medical University, Syracuse, USA; 2 Medicine, Guthrie Cortland Medical Center, Cortland, USA; 3 Medicine, State University of New York Upstate Medical University, Syracuse, USA; 4 Hematology and Oncology, University of Arizona, Tucson, USA; 5 Cardiology, State University of New York Upstate University Hospital, Syracuse, USA

**Keywords:** cardiac catheterization, cranial nerve palsy, double vision, neurological complications

## Abstract

Neurological complications after cardiac catheterization are rare. We report an unusual case of isolated third cranial nerve palsy in a 72-year-old male patient whose past medical history was significant for diabetes mellitus and coronary artery disease (CAD). He presented for elective cardiac catheterization for stable angina, which revealed multivessel CAD and no intervention was done. Two hours after the procedure, the patient suddenly started complaining of new-onset double vision in his left eye. Ophthalmologic exam revealed ptosis of the left eye lid, sluggish pupillary reflex and impaired adduction of the left eye along with exotropia of the left eye on primary gaze, all findings consistent with the left third nerve palsy. Rest of the neurological exam and neuroimaging (CT angiogram of head and MRI brain) were normal. Embolic phenomenon has been described as a possible mechanism in such patients leading to small vessel ischemic disease and cerebral microinfarction. Neuro-ophthalmologic complications after cardiac catheterization are rare but devastating for the patients. These should be recognized promptly, and patients should undergo neuroimaging to evaluate for any identifiable causes. These patients should be treated with aspirin and statin therapy and evaluated by ophthalmology for correction with prism lenses if symptoms persist.

## Introduction

Every year there are more than 1,000,000 cardiac catheterizations performed in the US [1]. Cardiac catheterization can lead to various complications, including neurological complications which are extremely rare and occur in less than 1% of the cases. Neurological complications include cerebrovascular events, including ischemic strokes, neuro-ophthalmological syndromes and peripheral neuropathies [2]. Although rare, the consequences of these neurological complications can be debilitating. Isolated cranial nerve palsy after cardiac catheterization is extremely rare, and we report an unusual case of isolated third cranial nerve palsy.

## Case presentation

We present a case of a 72-year-old male patient who presented for an elective cardiac catheterization for further evaluation of stable angina. His medical history was significant for diabetes mellitus and coronary artery disease (CAD) with a drug-eluting stent (DES) placed 16 years ago. He underwent cardiac catheterization through right radial artery approach which revealed multivessel CAD, no intervention was done and he was referred to cardiothoracic surgery team for evaluation of coronary artery bypass graft (CABG).

**Figure 1:**
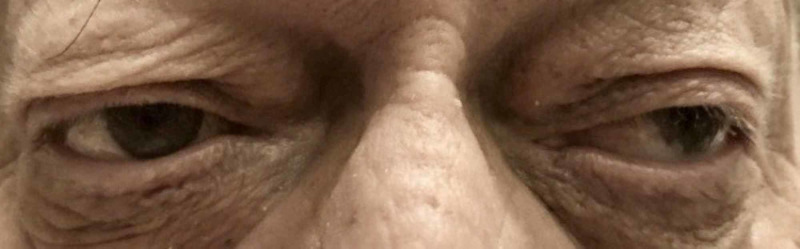
Exotropia of the left eye on primary gaze

**Figure 2:**
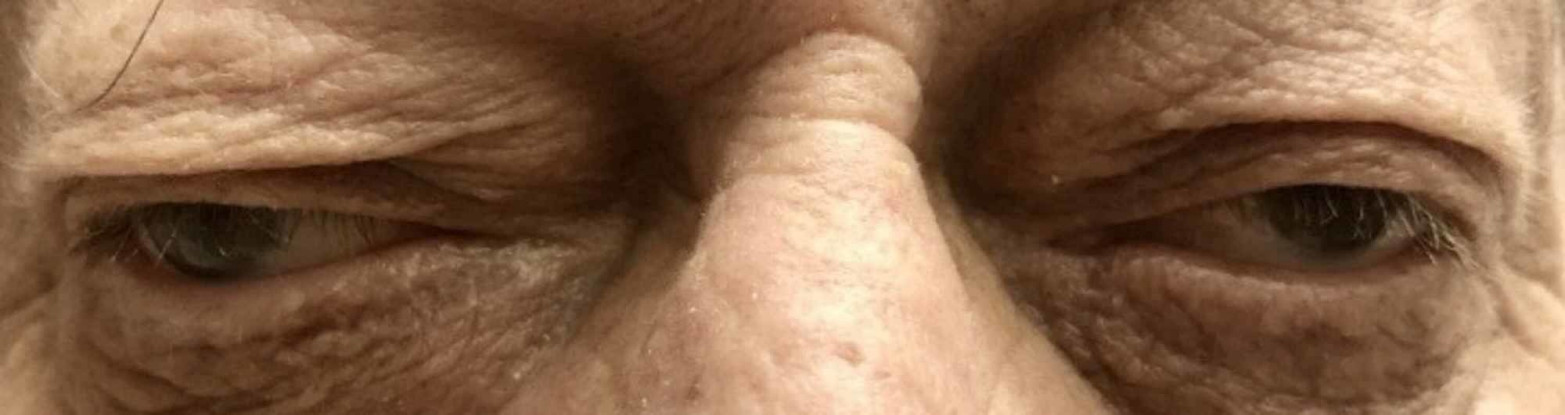
Impaired adduction of the left eye on rightward gaze

Two hours after the procedure, the patient suddenly started complaining of persistent visual disturbance and described it as seeing everything double; he had never experienced similar complaints in the past. He denied any headache, dizziness, speech difficulty or motor or sensory weakness in his extremities. The patient was hemodynamically stable and found to be in sinus rhythm. Ophthalmologic exam revealed mild ptosis of the left eyelid, sluggish pupillary reflex in the left eye and exotropia of the left eye on primary gaze along with impaired adduction of the left eye (Figures 1, 2).

**Figure 3:**
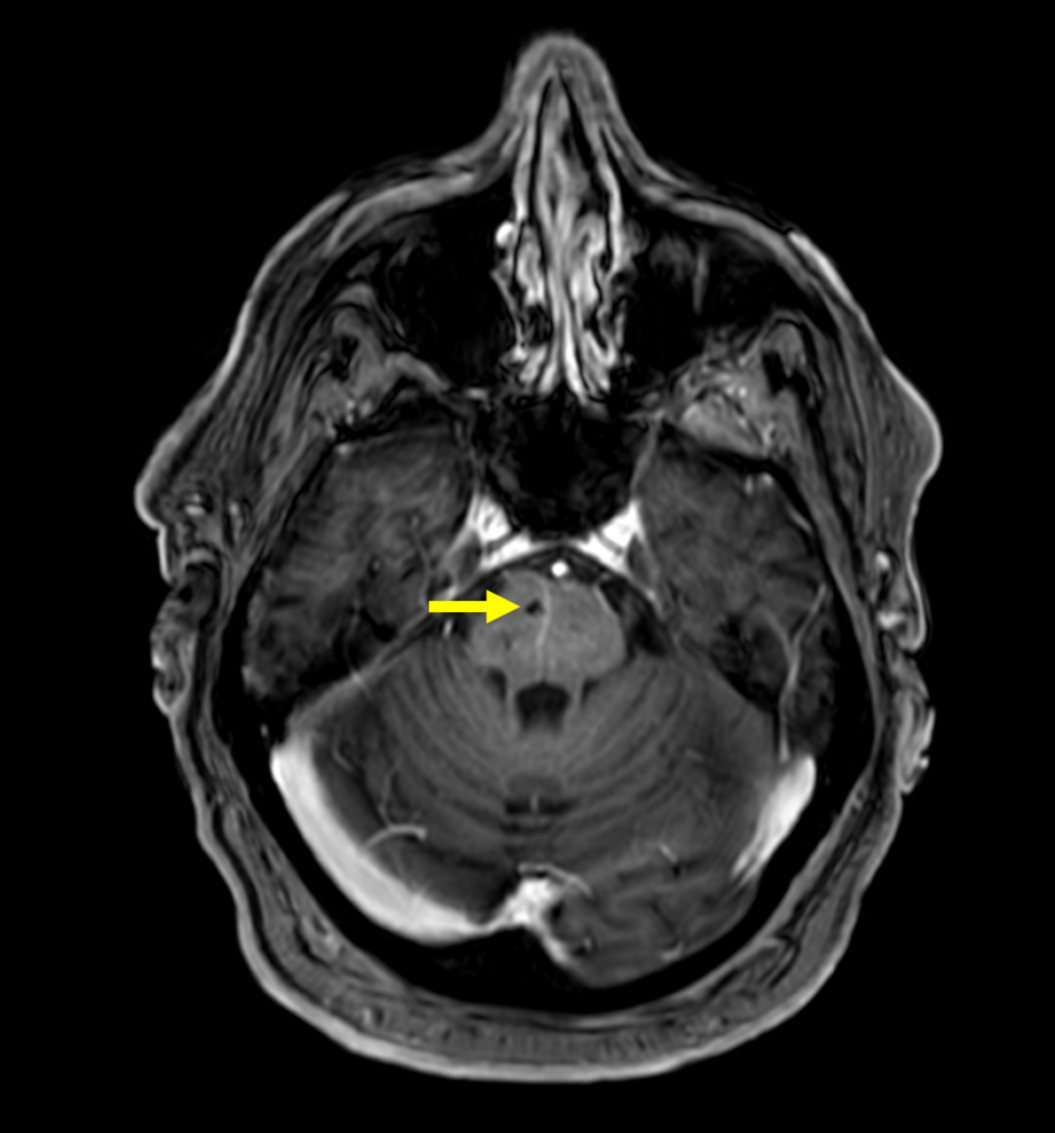
MRI of the brain showing small arteriovenous malformation in right pons (yellow arrowhead)

There were no abnormal findings in the right eye. The patient also complained of diplopia with downward and outward gaze, and these symptoms resolved transiently by covering his left eye. Rest of the exam for other cranial nerves was normal, and there were no focal motor or sensory deficits appreciated. Computed tomography angiography (CTA) of head and neck did not show any evidence of intracranial hemorrhage or territorial infarct, and revealed a small stable arteriovenous malformation (AVM) in right pons. MRI of the brain did not show any abnormal enhancement and third cranial nerve appeared normal bilaterally, and again demonstrated small cavernous malformation in right pons (Figure 3) similar to CT scan findings. He was already on atorvastatin and aspirin for his CAD which was continued.

The patient continued to have persistent diplopia during his hospital stay, underwent CABG and was discharged 10 days later in stable condition. The patient followed up in ophthalmology clinic in four weeks; at this time, he complained of same visual complaints and there was no change in exam findings compared to his discharge from the hospital described above. The patient again followed up in clinic eight weeks later and his symptom of diplopia had now completely resolved. Ophthalmologic exam revealed his left eye ptosis had resolved, left pupil was round and reactive to light, and adduction of the left eye was normal.

## Discussion

Our patient’s ophthalmologic exam findings were consistent with third cranial nerve palsy on initial presentation. Neuro-ophthalmological syndromes after cardiac catheterization are rare and to our knowledge, only one case of isolated third cranial nerve palsy has previously been reported in literature which resolved spontaneously within a few days.

The exact pathophysiology for neurological complications after cardiac catheterization remains unclear but embolism has been proposed as the primary mechanism, mainly cerebral embolism originating from large atherosclerotic vessels [3]. This hypothesis is supported by the findings of transcranial Doppler ultrasound during cardiac catheterization which revealed cerebral emboli [4]. Previous studies have suggested cerebral microinfarction and small vessel ischemic disease resulting from embolic phenomenon and imaging would be mostly normal in these cases, which seems to be the case in our patient with normal CTA head and MRI of the brain. Previously published data have suggested subclinical neurological injury in up to 15% of the patients undergoing cardiac catheterization [5]. Given the risk for cerebrovascular accident (CVA) after cardiac catheterization, any neurological symptoms should prompt emergent imaging, such as CT scan and MRI of the brain, because these can lead to long-term morbidity associated with the disease. Patient-related risk factors such as age, female sex, hypertension, diabetes mellitus and degree of atherosclerosis, especially in the coronary arteries, have been linked to higher chances of neurological complications in patients undergoing cardiac catheterization [6]. Additionally, the length of procedure and amount of contrast used are also associated with a higher risk [7]. Our patient had risk factors including old age, hypertension, diabetes mellitus and high atherosclerotic disease burden with multivessel CAD. In contrast to disease course of our patient, patient’s symptoms in the previously reported case resolved within few days which suggest transient vasospasm as another mechanism in addition to those described above. Given vascular involvement and proposed disease process similar to transient ischemic attack, these patients should be started on aspirin and statin therapy if they are already not on these medications. The patients should also be evaluated by ophthalmologist to rule out any other intrinsic ocular diseases which might need prompt intervention, especially those having risk factors like hypertension and diabetes which can lead to retinal involvement. Although most of these patients have spontaneous resolution of symptoms over the course of few weeks, they should routinely follow up with ophthalmologists. In case of persistent symptoms, these patients should be evaluated for prism lenses which can help improve their symptoms.

## Conclusions

Neuro-ophthalmologic syndromes after cardiac catheterization are extremely rare but can be devastating for the patients. Given preponderance for involvement of posterior cerebral circulation and risk for CVA, any neurological complaints including visual disturbance after cardiac catheterization should be promptly evaluated with appropriate imaging and followed up routinely for resolution of symptoms.
